# Co-sensitization of Copper Indium Gallium Disulfide and Indium Sulfide on Zinc Oxide Nanostructures: Effect of Morphology in Electrochemical Carbon Dioxide Reduction

**DOI:** 10.1021/acsomega.4c00018

**Published:** 2024-04-22

**Authors:** Cigdem Tuc Altaf, Tuluhan Olcayto Colak, Emine Karagoz, Jiayi Wang, Ya Liu, Yubin Chen, Maochang Liu, Ugur Unal, Nurdan Demirci Sankir, Mehmet Sankir

**Affiliations:** † Department of Materials Science and Nanotechnology Engineering, TOBB University of Economics and Technology, Sogutozu Caddesi No 43, Sogutozu 06560, Ankara, Turkey; ‡ Micro and Nanotechnology Graduate Program, TOBB University of Economics and Technology, Sogutozu Caddesi No 43, Sogutozu 06560, Ankara, Turkey; § International Research Center for Renewable Energy, State Key Laboratory of Multiphase Flow, Xi’an Jiaotong University, Xi’an, Shaanxi 710049, China; ∥ Department of Chemistry, Surface Science and Technology Centre (KUYTAM), Koç University, Rumelifeneri Yolu, 34450 Sariyer, Istanbul, Turkey

## Abstract

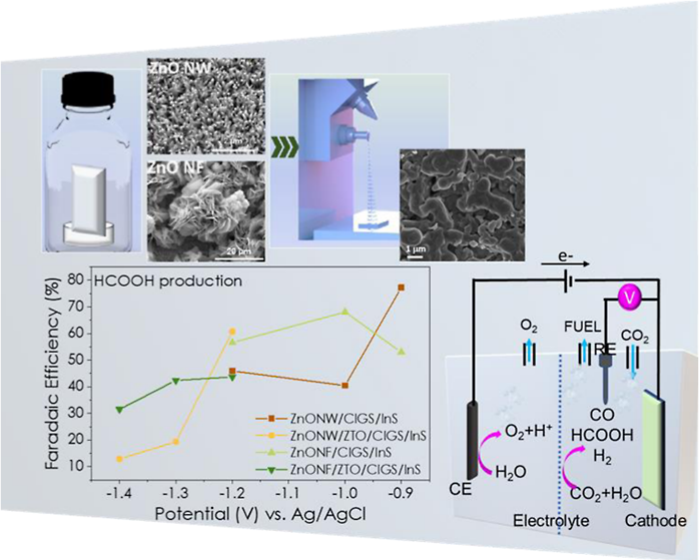

Recent advances in nanoparticle materials can facilitate the electro-reduction of carbon dioxide (CO_2_) to form valuable products with high selectivity. Copper (Cu)-based electrodes are promising candidates to drive efficient and selective CO_2_ reduction. However, the application of Cu-based chalcopyrite semiconductors in the electrocatalytic reduction of CO_2_ is still limited. This study demonstrated that novel zinc oxide (ZnO)/copper indium gallium sulfide (CIGS)/indium sulfide (InS) heterojunction electrodes could be used in effective CO_2_ reduction for formic acid production. It has been determined that Faradaic efficiencies for formic acid production using ZnO nanowire (NW) and nanoflower (NF) structures vary due to structural and morphological differences. A ZnO NW/CIGS/InS heterojunction electrode resulted in the highest efficiency of 77.2% and 0.35 mA cm^–2^ of current density at a −0.24 V (vs. reversible hydrogen electrode) bias potential. Adding a ZTO intermediate layer by the spray pyrolysis method decreased the yield of formic acid and increased the yield of H_2_. Our work offers a new heterojunction electrode for efficient formic acid production via cost-effective and scalable CO_2_ reduction.

## 1 Introduction

Converting carbon dioxide (CO_2_) into valuable chemicals and fuels has made an impact on reducing our carbon footprint.^1−3^ However, the high stability of CO_2_ for conversion into different chemicals restricts the application. Therefore, the research community has focused on developing materials and systems for efficient CO_2_ conversion by reducing the high activation energy of CO_2_.^3−7^ It is possible to achieve CO_2_ reduction via various routes, including photocatalytic and electrochemical conversion.^8−13^ In this way, besides decreasing carbon emissions, value-added chemicals, such as methanol, hydrogen, formic acid, and syngas, can be produced.^14−18^ Among these chemicals, formic acid stands out as an alternative to fossil fuels due to its advantages, such as being an energy-intensive material, having a high volumetric hydrogen density, and having enormous potential as an effective hydrogen storage vector.^19^ Typically, the most important factor in producing different types of chemicals, from formic acid to carbon monoxide and multicarbon hydrocarbons and oxygenates, is the selectivity of the used catalyst. At this point, it is inevitable to perform catalyst engineering to ensure product selectivity and enhanced efficiency. In other words, CO is generally the main product in the use of catalysts such as Zn, Ag, Au, and Pd, which are defined as Group I in the literature and thermodynamically support the adsorption of *COOH to *OCOH.^20^ Ga, In, Sn, and Bi belong to group II and often thermodynamically prefer *OCOH adsorption to *COOH, yielding formate as the main product. Group III, which has an ideal capacity to stabilize both *COOH and *CO, contains Cu-based catalysts and offers the possibility of multicarbon hydrocarbons and oxygenates product formation.^20,21^ Since Zn-based materials, which are group I members, come to the fore in CO production, different morphologies such as nanoplates, porous structures, or hexagonal structures have been used as catalysts in electrocatalytic CO_2_ reduction.^22,23^

Similarly, zinc oxide (ZnO) has been used as an electrocatalyst for CO production.^24,25^ On the other hand, heterogeneous catalysts have been designed to increase the Faradaic efficiency and reduce the overpotential.^26,27^ In the device-like configurations having the potential for large-scale fuel production, thin or free-standing film heterogeneous catalysts have started to get attention.^28−30^ In addition, the effect of the morphology of the transition-metal-based catalysts on electrochemical CO_2_ reduction has recently been investigated.^31−33^ ZnO has the advantage of the ease of synthesis in various morphologies for a great diversity of applications.^34,35^ In the designs with device structure and the potential to produce large amounts of fuel, studies on the development of thin-film heterogeneous catalysts grown on a conductive substrate have been favored.^28,29,36^ Therefore, there is a need for heterojunction thin-film catalysts with improved electrical and catalytic properties, with manufacturing techniques that can be applied to large areas. With this motivation, our group suggested the ZnO/copper indium gallium sulfide (CIGS)/indium sulfide (InS) heterojunction electrodes for electrochemical CO_2_ reduction.

Although there are some reports on the utilization of ZnO and metal sulfides for CO_2_ reduction via the electrochemical route,^37−40^ there are very limited reports on the copper-based chalcopyrite thin-film electrodes.^41,42^ Therefore, there is a strong necessity for the investigation of metal oxide/chalcopyrite/metal sulfide-based thin-film heterogeneous catalysts for electrochemical CO_2_ reduction. Previously, we demonstrated the efficiency enhancement in photoelectrochemical water splitting, in which CIGS/InS-based electrodes were used as the photoactive electrode.^34,43,44^ We also reported that the morphology control via precursor materials in the chemical bath produces nanostructures of ZnO that directly affect the performance of ZnO/CIGS/InS electrodes.^34^ Furthermore, the utilization of a zinc stannate, ZnSnO_3_ (ZTO), layer on hydrothermally grown 3D-ZnO nanostructures in between ZnO and CIGS layers provided better solar-to-hydrogen-conversion efficiency by enhancing the charge transfer in a photoelectrochemical system.^44^

In this study, the electrochemical reduction of CO_2_ to formic acid using ZnO electrodes with two different morphologies, sensitized with CIGS/InS, was investigated. In addition to formic acid production, it was also examined how the electrode structure used, different ZnO nanostructures, and the presence of a ZTO layer added between ZnO and CIGS by the spray pyrolysis method affected the production of CO and H_2_. Thus, we are pioneering the electrochemical production of formic acid by producing cost-effective electrodes, which are heterojunctions of nanostructures of metal oxide, chalcopyrite, and metal sulfide. In addition to all these, we believe that our study provides a detailed knowledge of the ZTO layer to obtain different reaction products. We believe that the electrochemical production of formic acid in higher yields and multiple amounts can be improved in the future by using the electrodes we have developed in this work.

## 2 Experimental Section

### 2.1 Electrode Preparation

**Figure 1:**
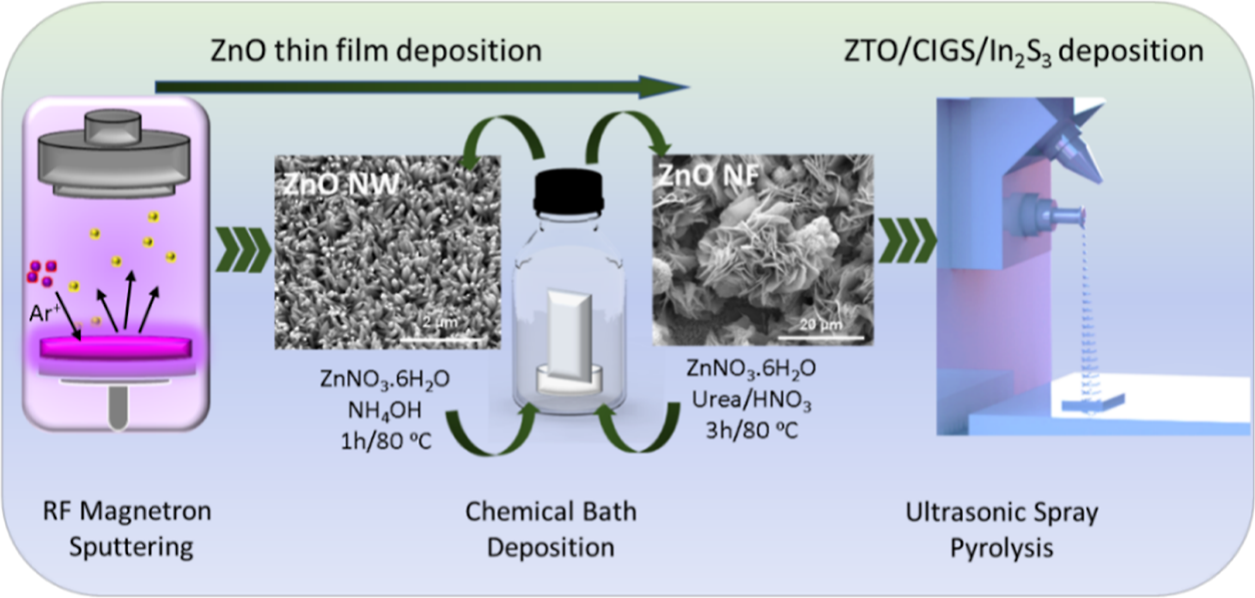
Schematic illustration of the ZnO/ZTO/CIGS/InS electrode preparation.

ZnO NW and NF thin films have been prepared via chemical bath deposition (CBD) as described in our earlier reports.^34,45^ ZTO and CIGS/InS layers have been deposited on the ZnO nanostructured thin films via ultrasonic spray pyrolysis.^46−49^ For the ZTO layer, 25 mM zinc acetate dihydrate (Zn(CH_3_COO)_2_·2H_2_O, Sigma-Aldrich, 99%) and 75 mM tin(IV) chloride pentahydrate (SnCl_4_·5H_2_O, Sigma-Aldrich, 98%) were dissolved in methanol–deionized water (v/v = 3:1) and sprayed on the ZnO thin films with a 20 pass number, 48 kHz at 200 °C. The ZTO layer was then annealed at 550 °C for 2 h for complete transformation of the ZnSn(OH)_6_ phase to ZTO. CIGS and In_2_S_3_ layers were deposited as described in the literature.^44^ The thin-film electrode preparation route is schematically summarized in Figure 1.

### 2.2 Electrochemical Measurements

The H-type electrochemical cell made from photosensitive resin with Teflon-coated silicon o-rings has been used for electrochemical CO_2_ reduction experiments. The prepared thin films have been employed as cathodes, while Pt sheet (thickness of 1 mm) and Ag/AgCl have been used as the counter and the reference electrodes, respectively. All of the components have been soaked in 0.1 M HNO_3_ for surface cleaning before use. An anion exchange membrane (FUMASEP, FAA-3-PK-130) was used to separate the working and the counter electrode compartments and to prevent the oxidation of reduced CO_2_ products. The cell was designed to have a certain electrode area (1 cm^2^) and a small electrolyte volume (3 mL) in each of the two compartments. CO_2_ (5 N), regulated by a mass flow controller (Alicat MC-50SCCM-D) at 5 sccm, flowed through the cell during electrolysis. A 0.1 M solution of KHCO_3_ (Sinopharm Chemical Reagent Co., Ltd.) was prepared by using 18.2 MΩ deionized water from a Millipore system as the electrolyte. The pH of the electrolyte purged with CO_2_ was 6.8.

All electrochemical data was collected vs a leak-free Ag/AgCl reference electrode and converted to a reversible hydrogen electrode (RHE) scale by the following equation: *V*_vsRHE_ = *V*_vsAg/AgCl_ + 0.259 V + 0.059 V × 6.8_(pH of soln)_. Autolab (Metrohm, PGSTAT302N) software was used to link different techniques for each electrolysis experiment. Aliquots of the reactor exhaust were injected via an automated sample loop into a gas chromatograph (GC, Agilent 7890). Aliquots were collected after 6, 16, 26, and 36 min of chronoamperometry to determine the concentration of gaseous products. The current efficiency was calculated by determining the number of coulombs needed to produce the measured amount of each product and then dividing it by the total charge passed during the time of GC sampling.

## 3 Results and Discussion

### 3.1 Materials Characterization

**Figure 2:**
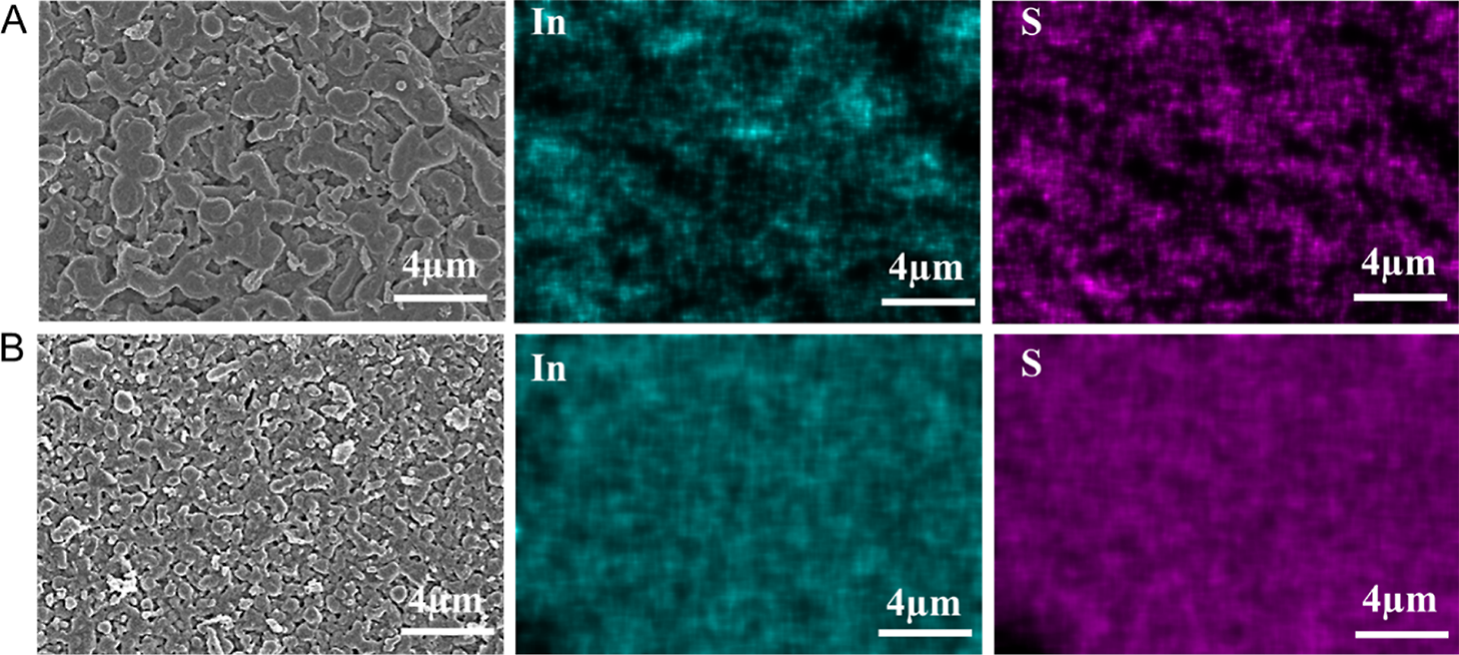
SEM images and EDS mapping of (A) ZnO NW/ZTO/CIGS/InS and (B) ZnO NF/ZTO/CIGS/InS thin-film electrodes.

CIGS/InS electrodes have been deposited on two different ZnO nanostructures, namely, nanowires (NWs) and nanoflowers (NFs). The SEM images at high and low magnifications are given in Figure S1, indicating the formation of flower- and wire-like structures for NFs and NWs. In our previous studies, we have demonstrated the influence of reaction conditions and types of precursors on the resulting morphologies.^34,50−52^ The acidic medium in the CBD solution resulted in flower-like morphologies, whereas the basic solution led to the formation of wire-like ZnO nanostructures. It is known that precipitates of zinc complexes (Zn(OH)_2_ and Zn(OH)_4_^2+^) form as the zinc salts react with hydroxyl (OH^–^) ions in the reaction medium. In the first stage of this process, Zn(OH)_2_ precipitate forms when Zn ions react with OH^–^ ions. As the OH^–^ ion concentration increases in the solution, the Zn(OH)_2_ precipitate dissolves to form a homogeneous solution containing Zn(OH)_4_^2–^ ions. A highly crystallized ZnO powder is obtained by heating this aqueous solution containing Zn(OH)_4_^2–^ ions at temperatures above 75 °C. Variation of morphology occurs when the pH of the medium changes. The basic reaction medium for ZnO NWs with excess OH^–^ ion concentration leads to the ZnO growth in one direction (*c*-axis). On the contrary, in a slightly acidic medium (pH 4.8–5.4), excess H^+^ ions react with OH^–^ ions at the surface and inhibit the growth in the *c*-axis, leading to the formation of sheet-like nanostructures.^53^ Thus, by altering the conditions in the reaction medium, a diversity of ZnO nanostructures can be achieved on the basis of the direction of the growth rate. After spray pyrolysis of ZTO, CIGS, and InS layers on ZnO nanostructures, the surface topography was observed to be homogeneous, completely covering the underlying layer (Figure 2). While the ZnO NWs had larger surface agglomerations (Figure 2A), the coatings on the ZnO NFs resulted in smaller agglomerations (Figure 2B). In addition, it was determined that indium and sulfur belonging to the uppermost layer were homogeneously distributed on the surfaces of the samples. It was verified that the ZTO thin film coated between the ZnO nanostructure and CIGS layers did not cause significant differentiation in the surface morphology. In addition, SEM images for the spray-pyrolyzed ZTO layer on the bare FTO, ZnO NW, and ZnO NF are given in Figures S2–S4, respectively.

**Figure 3:**
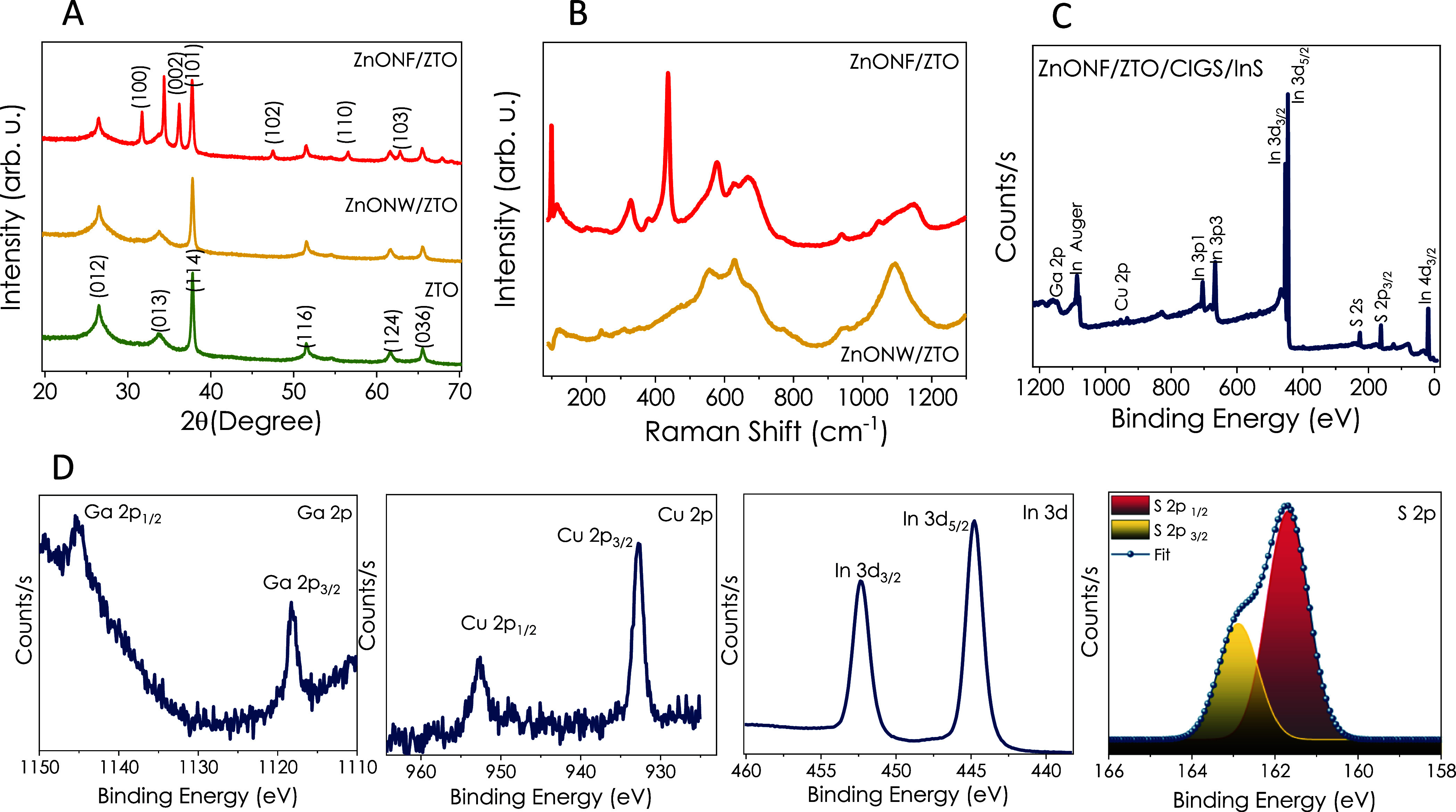
(A) XRD, (B) Raman spectra of ZTO-coated ZnO nanostructures, (C) XPS survey spectrum, and (D) high-resolution XPS spectra of ZnO NF/ZTO/CIGS/InS.

The structures of the ZTO-coated ZnO NW and NF samples have been confirmed via X-ray diffraction (XRD) and Raman spectroscopies (Figures 3A,B). Figure 3A displays the XRD pattern of ZTO-decorated ZnO NW and NF thin films along with the pristine ZTO thin film. The diffraction peaks residing at 26.47, 33.74, 37.83, 51.50, 61.55, and 65.47° are well indexed to the crystal planes of (012), (013), (114), (116), (124), and (036), respectively, matching with the orthorhombic crystal phase of ZnSnO_3_, as in previously reported works (JCPDS: 28-148).^44,54,55^ The absence of the characteristic ZnSn(OH)_6_ phase at 22° (200) indicated a complete transformation of ZnSn(OH)_6_ to ZnSnO_3_ after calcination at 550 °C. As reported in our previous work, the formation of ZnO NW and NF nanostructured thin films varies with the types of precursors (urea and NH_4_OH) and pH in the hydrothermal reaction solution.^45,53,56,57^ However, both conditions result in hexagonal wurtzite ZnO formation as presented in the XRD spectra of the thin-film heterojunctions (JCPDS card no. 36-1451).^44^ After spray pyrolysis of the ZTO layer, the XRD pattern of ZnO NF displays diffraction peaks belonging to the characteristic hexagonal wurtzite crystal structure. However, it was observed that the ZTO layer completely suppressed the XRD signals of ZnO NW, possibly due to the narrower thickness of the ZnO NW thin film having ∼2.0 μm length NWs.

It is known that the Raman signals of ZnO are usually sensitive to the structure of crystals as well as to the defects in the crystal structure.^44,58^ Raman shifts at around 100 cm^–1^ wavenumbers were more pronounced for the ZnO NF/ZTO sample compared with ZnO NW/ZTO. Previously, Raman shifts between 100 and 280 were assigned to the E_2_ low and Zn–Zn disorder.^59,60^ In addition, one of the important differences between the Raman spectra of these two samples is that the sharp peaks seen at 335 and 438 cm^–1^ in NF films are not so evident in the NW spectrum. In the literature, peaks around 330 cm^–1^ are assigned to the second-order Raman spectrum arising from zone-boundary phonons 3E_2H_–E_2L_.^58^ The sharp peak in the ZnO NF/ZTO sample at 438 cm^–1^ is the characteristic E_2_ high mode, typical to the hexagonal phase of ZnO.^61^ The absence of this peak for the ZnO NW/ZTO sample is evidence of a structural change. Moreover, the broad bands E_1_-LO and A_1_-LO ranging from 520 to 590 cm^–1^ are shown in both samples. A combination of acoustical and optical modes is also shown in both samples around 670 cm^–1^.^60^ Finally, it has been observed that both samples have multiphonon modes corresponding to the A_1_ + E_2_ symmetry around 1100 cm^–1^.^62,63^ Additionally, the Raman spectrum of the ZTO layer itself has been characterized in Figure S5.

Figure 3C displays the survey spectrum of the ZnO NF/ZTO/CIGS/InS electrode, indicating the main signals of the In_2_S_3_ and CIGS layers. In the spectrum, In 4d, Ga 3d, Cu 2p, and Ga 2p core-level XPS peaks are in good agreement with the previous works.^44,64^ High-resolution XPS analysis results obtained from the surface are given in Figure 3D. The high-resolution XPS peaks in the Ga 2p region appear at 1145.12 and 1118.47 eV for Ga 2p_1/2_ and Ga 2p_1/2_, respectively. In addition, the two signals in the Cu 2p region at higher (952.71 eV) and lower BE (932.67 eV) with 20.04 eV of spin–orbit splitting confirm the Cu^+^ ion in the CIGS compound. Since Cu^+^ and Cu^0^ states cannot be distinguished from the Cu 2p spectrum, we have also obtained the CuLMM spectrum, as given in Figure S6. The Cu^2+^ state gives strong satellite peaks in the 940–944 eV range in the Cu 2p spectrum. The missing peaks in the Cu 2p spectra for our samples provide evidence that there is no Cu^2+^ state. The peak at 595 eV can be assigned to the Cu^+^ state. There is a very weak peak at 566.6 eV, which can be assigned to the Cu^0^ state. Thus, we may conclude that the sample mainly consists of the Cu^+^ state with a small amount of the Cu^0^ state.^65,66^ In 3d signals (at 444.78 and 452.34 eV for In 3d_5/2_ and In 3d_3/2_, respectively) are in good agreement with those of In^3+^. The S 2p peak can be deconvoluted into two Gaussian peaks, residing at 161.49 and 162.79 eV and are evidence of the presence of a S^2–^ ion. Additional XPS analysis results are given in Figures S6–S9 for ZnO NW/CIS/InS, ZnO NW, and ZTO layers.

**Figure 4:**
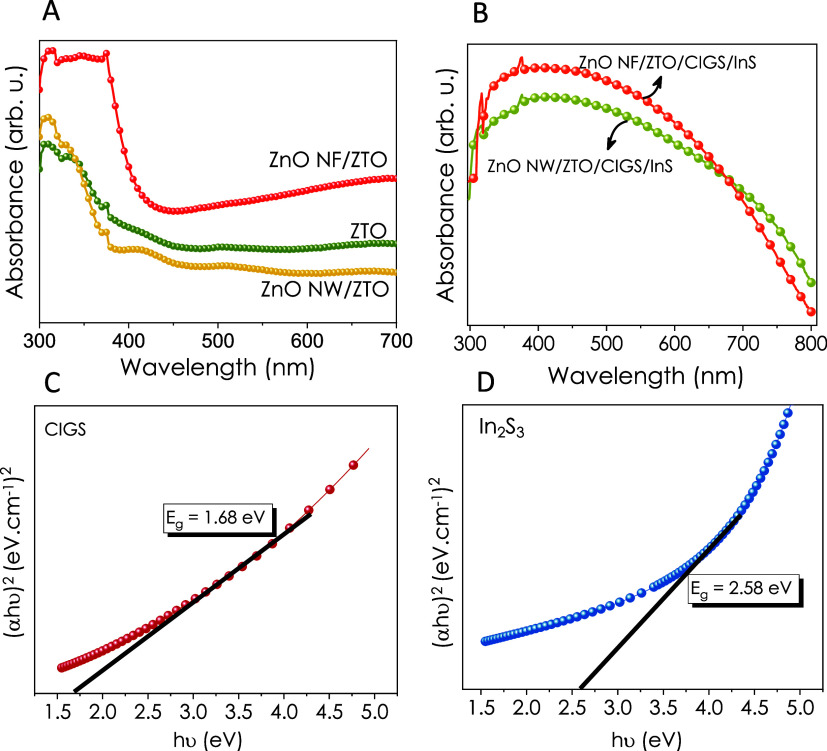
UV–vis spectra of (A) metal oxide heterostructures and (B) CIGS/InS-decorated metal oxide samples; Tauc plots of spray-pyrolyzed (C) CIGS and (D) InS thin films.

Optical characterization of the thin-film samples was performed using UV–vis spectroscopy (Figure 4) to observe the changes in the absorption ranges. Absorption of ZnO NF/ZTO was higher than both bare ZTO and ZnO NW/ZTO in wavelengths between 300 and 700 nm. Also, as expected, by the inclusion of the CIGS and InS layers absorption was shifted through the visible region. The optical band gaps of CIGS and InS thin films calculated from Tauc plots were 1.68 and 2.58 eV, respectively.

### 3.2 Electrochemical CO_2_ Reduction Analysis

**Figure 5:**
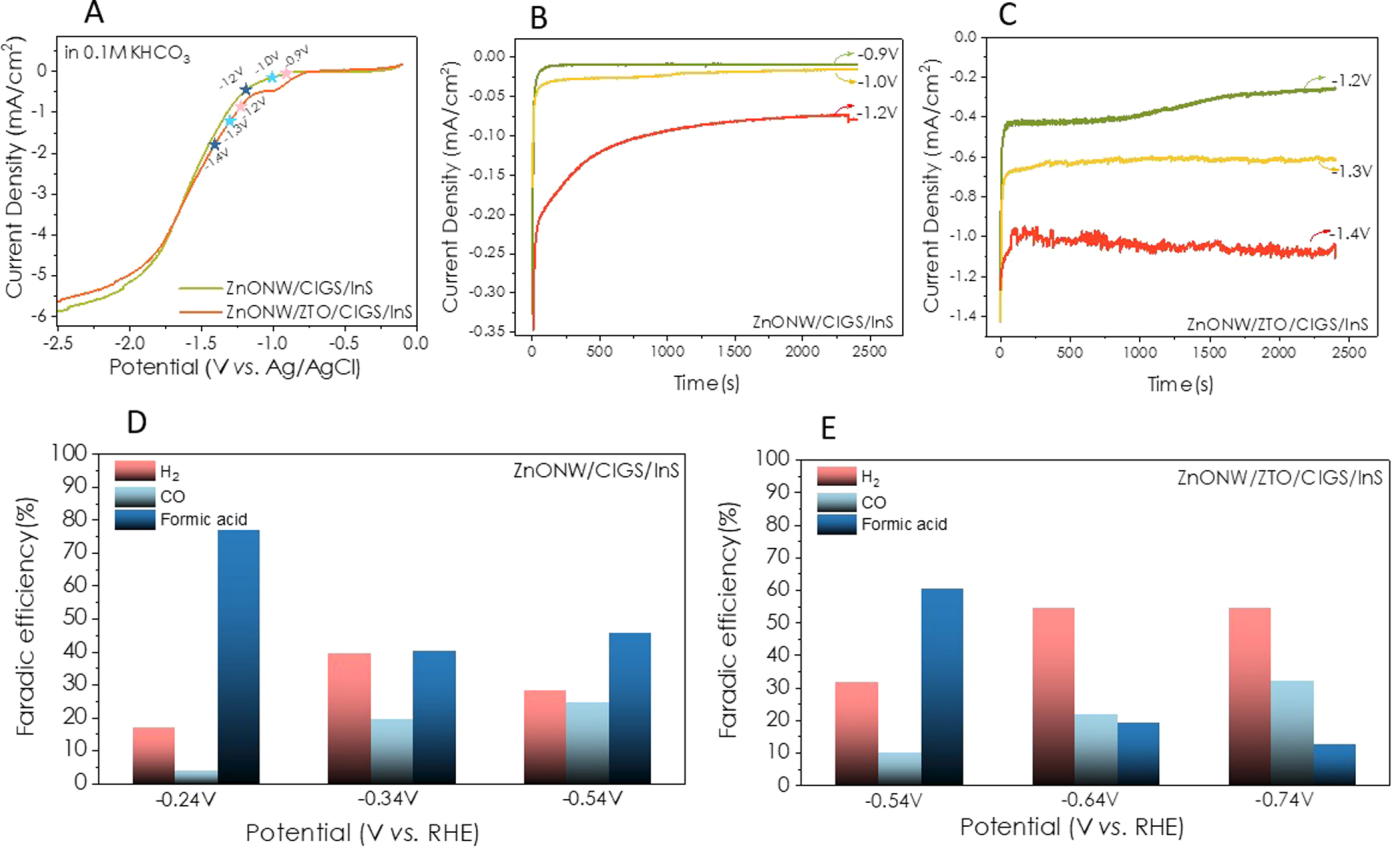
Electrochemical CO_2_ reduction tests of ZnO NW/CIGS/InS and ZnO NW/ZTO/CIGS/InS thin-film electrodes (A) LSV plots, (B,C) current density vs. time plots, and (D,E) Faradaic efficiencies for H_2_, CO, and formic acid products.

The electrochemical performances of CIGS/InS-coated ZnO NW thin films depicted that formic acid, H_2_, and CO were the main products. The linear sweep voltammetry (LSV) conducted in 0.1 M KHCO_3_ revealed the high current density over the potential window between 0 and −2.5 V (vs. Ag/AgCl) (Figure 5A). The stability of electrodes has been enhanced after ZTO coating (Figure 5B,C). Although there was a sharp decrease in the current density at the initial time interval, superior current densities were obtained with the ZTO interlayer. The current density of −1.0 mAcm^–2^ was stable over 2500 s at −1.4 V (vs. Ag/AgCl) for the ZnO NW/ZTO/CIGS/InS electrode. This increase in stability can be attributed to the very slow recombination of electron–hole pairs in the process as evidenced in our previously reported work.^44^ The operating potential has been converted to RHE, and FE % values have been evaluated (as depicted in Figure 5D,E) for both the ZnO NW/CIGS/InS and ZnO NW/ZTO/CIGS/InS electrodes, respectively.

**Figure 6:**
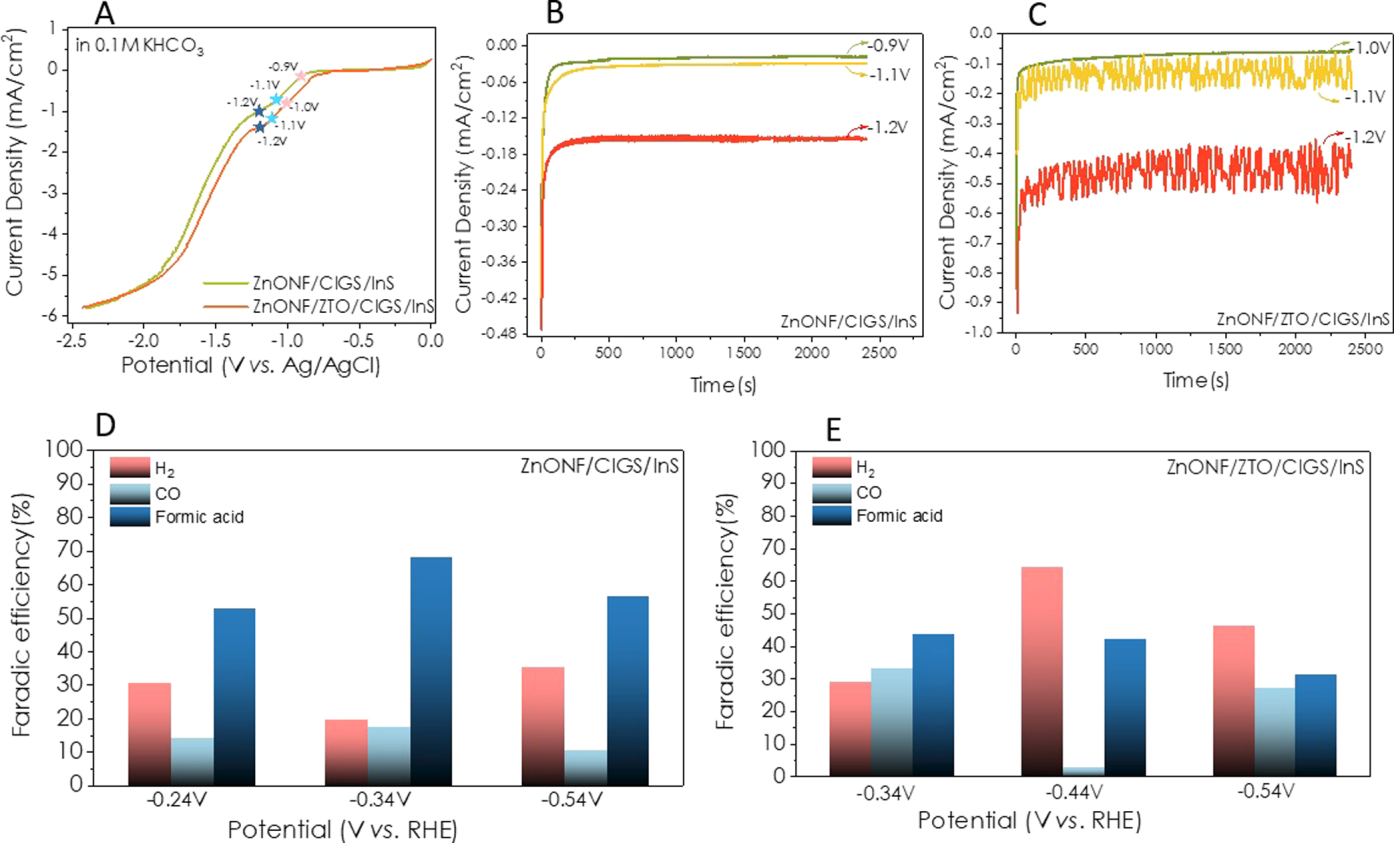
Electrochemical CO_2_ reduction tests of ZnO NF/CIGS/InS and ZnO NW/ZTO/CIGS/InS thin-film electrodes (A) LSV plots, (B,C) current density versus time plots, and (D,E) Faradaic efficiencies for H_2_, CO, and formic acid products.

On the other hand, the maximum Faradaic efficiency of 77.2% was calculated for formic acid production using the ZnO NW/CIGS/InS electrode. After ZTO deposition, this performance decreased to 62%, while the Faradaic efficiency of H_2_ production increased from 15 to 35%. Besides, with the ZTO layer, a maximum of 55% efficiency of H_2_ production was obtained for a −1.4 V (vs. Ag/AgCl) bias. Through the addition of the ZTO layer on the ZnO nanostructure, the H_2_ yield increased, while the decrease in formic acid production could be explained by the shift of the conduction band edge of the formed heterojunction electrodes to around 0 V compared to NHE.^44^ A similar trend has been observed for the ZnO NF/CIGS/InS and ZnO NF/ZTO/CIGS/InS electrodes (Figure 6). In other words, in ZnO NF electrodes, like ZnO NW electrodes, the addition of the ZTO intermediate layer by the spray pyrolysis method decreases the yield of formic acid while increasing the yield of H_2_. Also, if we compare these two ZnO morphologies, then ZnO NW/CIGS/InS electrodes showed the best performance in formic acid production, while ZnO NF/ZTO/CIGS/InS showed the best performance in H_2_ production.

**Figure 7:**
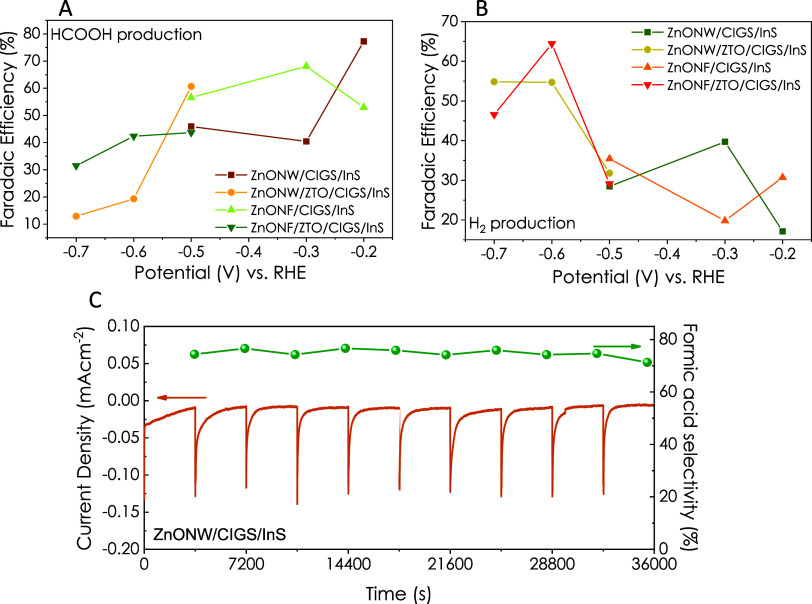
Faradaic efficiency versus bias potential for (A) formic acid and (B) H_2_ production; (C) long-term stability of ZnO NW/CIGS/InS electrode.

Furthermore, in most of the bias potentials, the CO production efficiency was lower than the Faradaic efficiency of formic acid (Figures 5 and 6). To better understand and compare the two different morphologies and effects of the ZTO layer, Faradaic efficiency for formic acid and H_2_ dependent on the bias potential is given in Figure 7. The ZnO NW/CIGS/InS heterojunction electrode resulted in the highest efficiency of 77.2% at a −0.24 V (vs. RHE) bias potential. On the other hand, the ZnO NF/ZTO/CIGS/InS heterojunction electrode resulted in higher H_2_ production at a −0.64 V (vs. RHE) bias potential compared to those of the other electrode configurations and potentials. Figure 7C displays the stability of the ZnO NW/CIGS/InS electrode for a 10 h duration, indicating high stability in terms of selectivity and current density.

**Figure 8:**
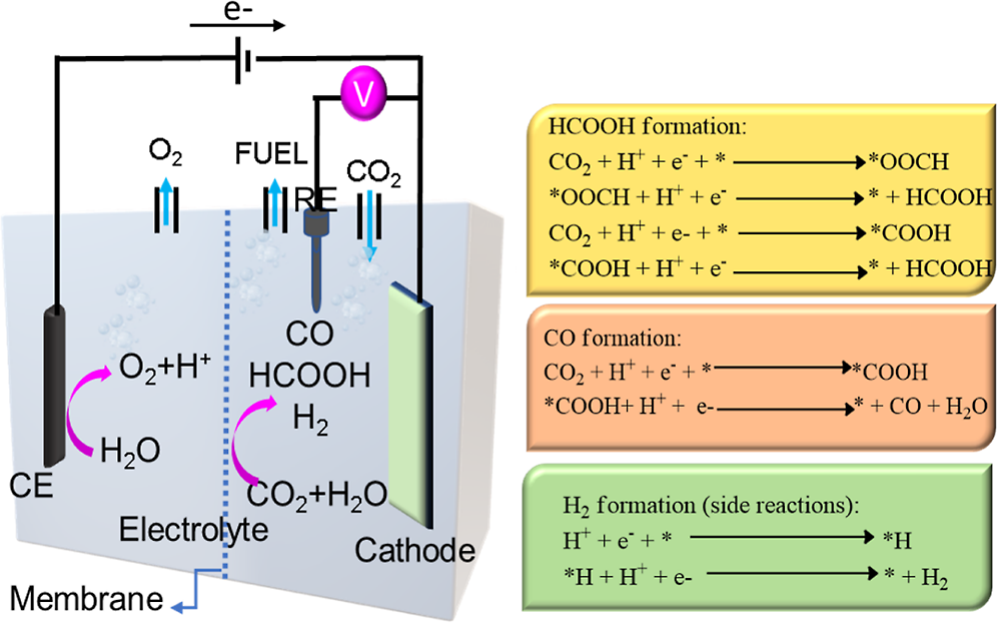
Schematic illustration of the electrochemical CO_2_ reduction system and the mechanisms to produce valuable chemicals from electrochemical CO_2_ reduction.

Figure 8 displays the schematic of the electrochemical-CO_2_ cell used in this work and the mechanisms for the electrochemical CO_2_ reduction into valuable products, such as H_2_, HCOOH, and CO, that takes place with the intermediate steps reported previously.^67,68^ The valence band positions of ZnO NW and NF thin films have been determined via XPS analysis as 3.23 and 2.81 eV, respectively (Figure S10). On the other hand, valence and conduction band positions of CIGS and In_2_S_3_ layers have been reported in our previous studies.^44^ The mechanism of formate/formic acid formation from CO_2_ is not yet fully comprehended. Still, it has been explained that the mechanism includes three main stages comprising the adsorption of the reactant on the surface of the electrode, the transfer of protons and electrons, and finally the desorption of the products from the electrode. In the mechanism, it was reported that the pores and surface area of the electrode material affect the electro-reduction performance toward formate formation.^69^ Shortly, as the porosity increases, the current density of formate increases. Therefore, the catalyst should be mesoporous to enhance the contact surface area between the electrolyte and the electrode. In our study, when comparing the CIGS/InS-coated ZnO NF- and NW-based electrodes at −1.2 V potential, the ZnO NF/CIGS/InS electrode exhibited a higher current density because of the higher specific surface area. In terms of selectivity, surface charge plays an important role in enhancement by generating new intermediates and stabilizing the chemical interactions with the catalyst.^70^

As a cathode, ZnO/CIGS/InS electrodes have a proper band edge position for the electrochemical formic acid production. Besides, shifting the conduction band through more positive potential values via the ZTO interlayer provides more favorable reactions for H_2_ generation. To compare the performance of our electrodes with the existing literature, we summarized the Faradaic efficiencies of copper-based chalcopyrite and chalcogenide electrodes reported so far (Table 1). As can be seen, our electrodes resulted in one of the highest Faradaic efficiencies for HCOOH production in the literature.

**Table 1:** Performance Summary of Chalcogenide-Based Electrodes for Electrocatalytic CO_2_ Reduction to Formic Acid/Formate Ion

cathode	*E* (V)	FE (%)	methods for electrocatalyst	electrolyte	ref.
ZnO NW/CIGS/InS	–0.24 V vs. RHE	77.2% [HCOOH]	CBD/spray pyrolysis	0.1 M KHCO_3_	this work
ZnO NF/CIGS/InS	–0.24 V vs. RHE	68.1% [HCOOH]			
ZnO/g-C_3_N_4_	–0.934 V vs RHE	80.99% [HCOO^–^]	hydrothermal	0.5 M KHCO_3_	(71)
In_2_S_3_	–1.4 V vs. RHE	20% [HCOOH]	hydrothermal	0.5 M KHCO_3_	(72)
Cu foil/CuS_*x*_	–1.2 V vs. RHE	69.1% [HCOOH]	electroplating	H_2_S-purged 0.1 M KHCO_3_	(73)
Ag@ZnO@rGO	–1.6 V vs. SCE	70% [HCOOH]	hydrothermal	0.5 M KHCO_3_	(74)
Cu pillars	–0.5 V vs. RHE	28.0% [HCOOH]	electrodeposition	0.1 M KHCO_3_	(75)
Cu/Cu_2_O/3VG	–1.0 V vs. RHE	49.8% [HCOOH]	PECVD/galvanostatic PED	0.1 M KHCO_3_	(76)

## 4 Conclusions

In this work, novel electrodes were produced for electrochemical-CO_2_ reduction application by the spray coating of copper-based chalcopyrite CIGS and sulfur-based chalcogen InS thin films on two different ZnO nanostructures, nanowires and nanoflowers. In addition, the effects of the spray-pyrolyzed ZTO film layer between the ZnO nanostructure and CIGS on the electrochemical performance were investigated. The structural differences between ZTO-coated ZnO NW and ZnO NF nanostructures were determined by Raman and XRD analysis. XPS analysis results confirmed the surface composition and valence band positions. Also, band gap and absorption edge shift obtained from UV–vis spectra evidenced the successful deposition of CIGS and InS materials on ZnO nanostructures. While electrochemical measurements confirmed that the more efficient electrode was ZnO NW/CIGS/InS thin-film electrode for formic acid formation, the efficiency values decreased with the addition of ZTO between ZnO and CIGS layers. On the other hand, it was observed that H_2_ production was more favorable for the electrodes with the ZTO interlayer. ZnO NW/CIGS/InS heterojunction electrode resulted in the highest efficiency of 77.2% at a −0.24 V (vs. RHE) bias potential. On the other hand, the ZnO NF/ZTO/CIGS/InS heterojunction electrode resulted in higher H_2_ production at a −0.64 V (vs. RHE) bias potential compared to the other electrode configurations and potentials. This study will contribute to the production of more efficient and cost-effective electrodes for electrochemical-CO_2_ reduction for formic acid formation in the future using scalable production methods such as spray pyrolysis and CBD.

## Supporting Information Available

SEM images of ZnO NW and NF thin films, SEM and EDS spectrum of ZTO thin film, SEM images of ZnO NW/ZTO thin film, SEM images of ZnO NF/ZTO thin film, Raman spectrum of ZTO layer, CuLLM region obtained from XPS spectra of ZnO NW/CIGS/InS, XPS spectra of ZnO NW/CIGS/InS, XPS spectra of ZnO NW, XPS spectra of ZTO, and valence band position of ZnO thin films (PDF)
