# Supplementary material for: Co-sensitization of Copper Indium Gallium Disulfide and Indium Sulfide on Zinc Oxide Nanostructures: Effect of Morphology in Electrochemical Carbon Dioxide Reduction
Source: ACS Omega. 2024 Apr 22;9(17):19209–18. doi: 10.1021/acsomega.4c00018 (PMC11064200; doi:10.1021/acsomega.4c00018)
Supplement: Supplementary file 1 — ao4c00018_si_001.pdf [file ao4c00018_si_001.pdf]

Supporting Information

Co-Sensitization of Copper Indium Gallium Disulfide and Indium Sulfide on Zinc Oxide Nanostructures: Effect of Morphology in Electrochemical Carbon Dioxide Reduction

Cigdem Tuc Altaf [a], Tuluhan Olcayto Colak, [b] Emine Karagoz, [b] Jiayi Wang, [c] Ya Liu, [c] Yubin Chen, [c] Maochang Liu, [c], Ugur Unal, [d] Nurdan Demirci Sankir, [a,b] Mehmet Sankir, [a,b]

[a] Department of Materials Science and Nanotechnology Engineering, TOBB University of Economics and Technology, Sogutozu Caddesi No 43 Sogutozu 06560 Ankara, Turkey

[b] Micro and Nanotechnology Graduate Program, TOBB University of Economics and Technology, Sogutozu Caddesi No 43 Sogutozu 06560 Ankara, Turkey

[c] International Research Center for Renewable Energy, State Key Laboratory of Multiphase Flow, Xi'an Jiaotong University, Xi'an, Shaanxi 710049, China

[d] Department of Chemistry, Surface Science and Technology Centre (KUYTAM), Koç University, Rumelifeneri Yolu, 34450 Sariyer, Istanbul, Turkey

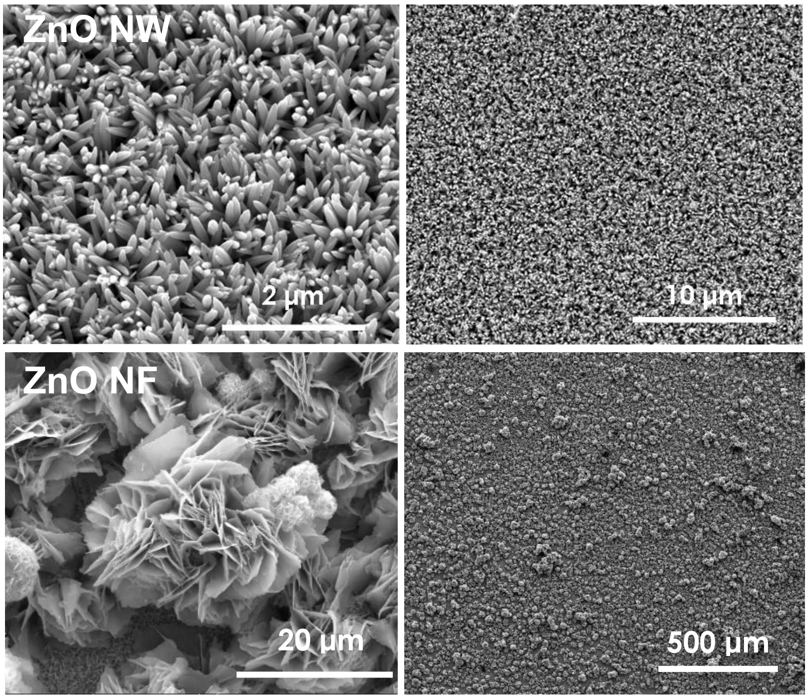

Figure S1. SEM images of ZnO NW and NF thin films

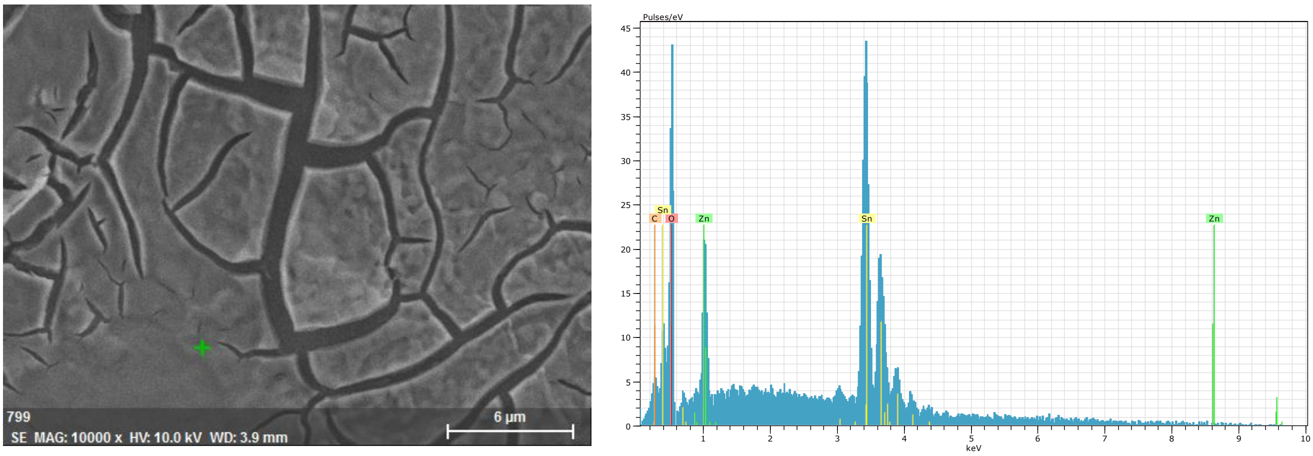

Figure S2. SEM and EDS spectrum of ZTO thin film

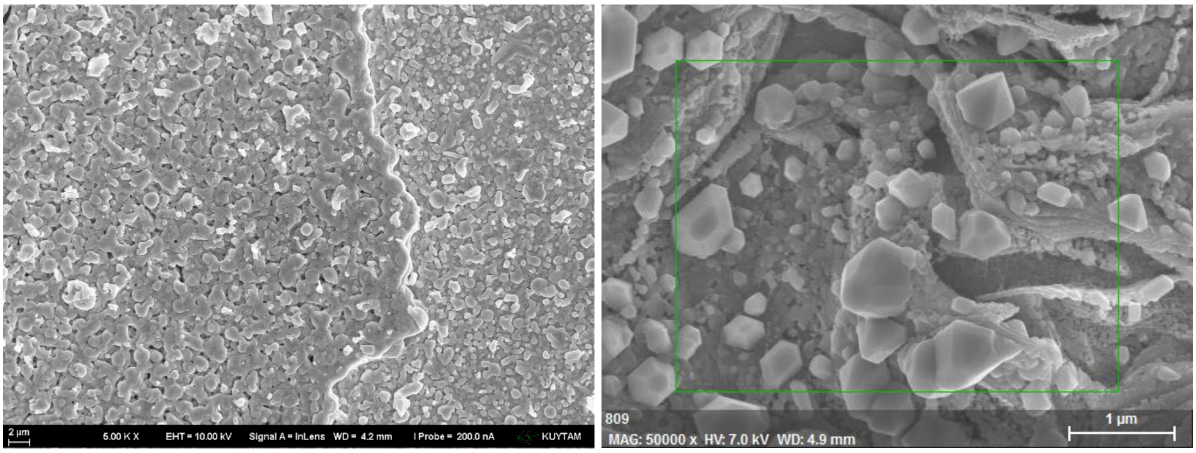

Figure S3. SEM images of ZnO NW/ZTO thin film

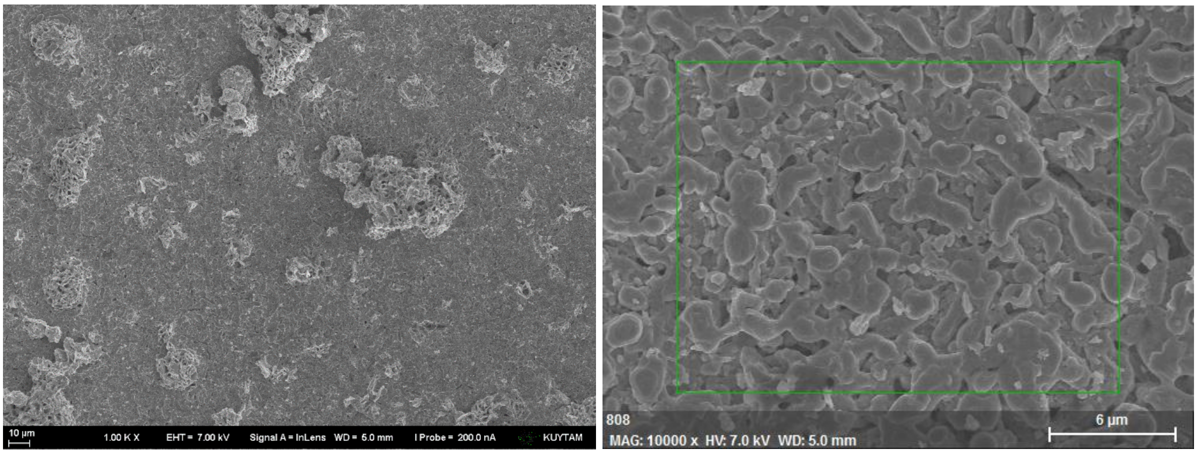

Figure S4. SEM images of ZnO NF/ZTO thin film

Figure S5 displays the Raman spectra of the ZTO layer annealed at 550 °C. The observed Raman modes can be seen at 308 and 474 cm^{-1} representing the E(LO) and A1(LO) symmetries, respectively. The broad peak embracing 564 and 639 cm^{-1} modes corresponds to the characteristic Raman shift of $ZnSnO_3$ of the stretching vibration mode of M-O.

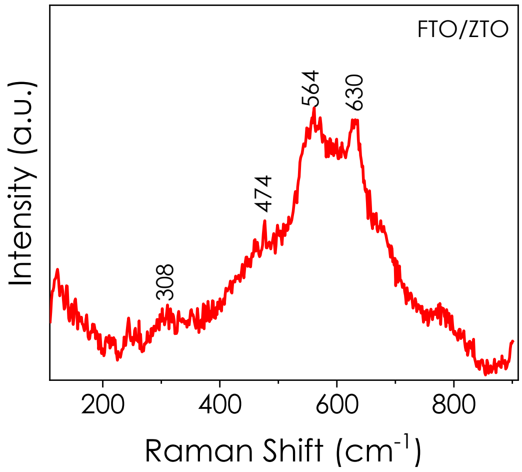

Figure S5. Raman spectrum of ZTO layer.

Additional XPS analysis of the thin films

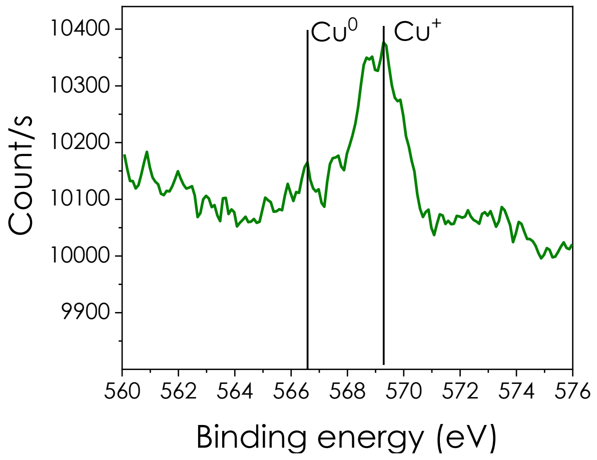

Figure S6. CuLLM region obtained from XPS spectra of ZnONW/CIGS/InS

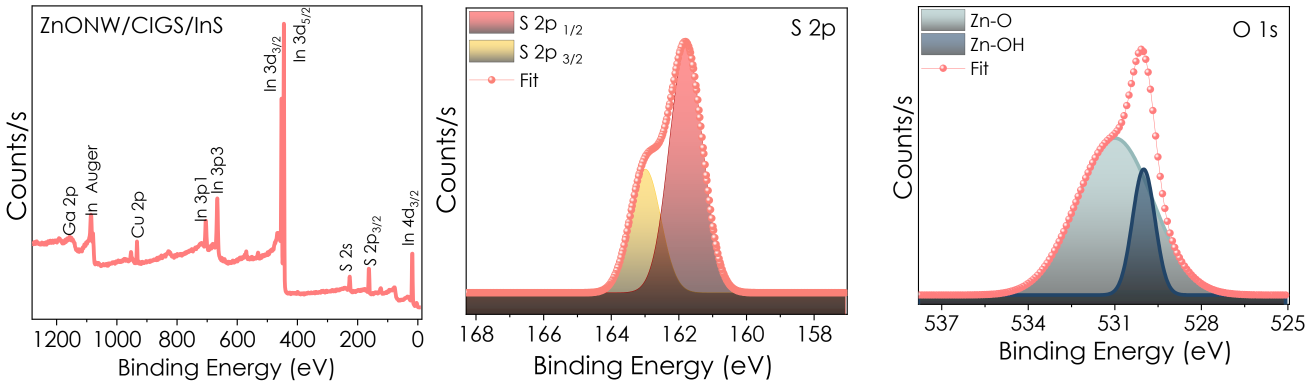

Figure S7. XPS spectra of ZnONW/CIGS/InS

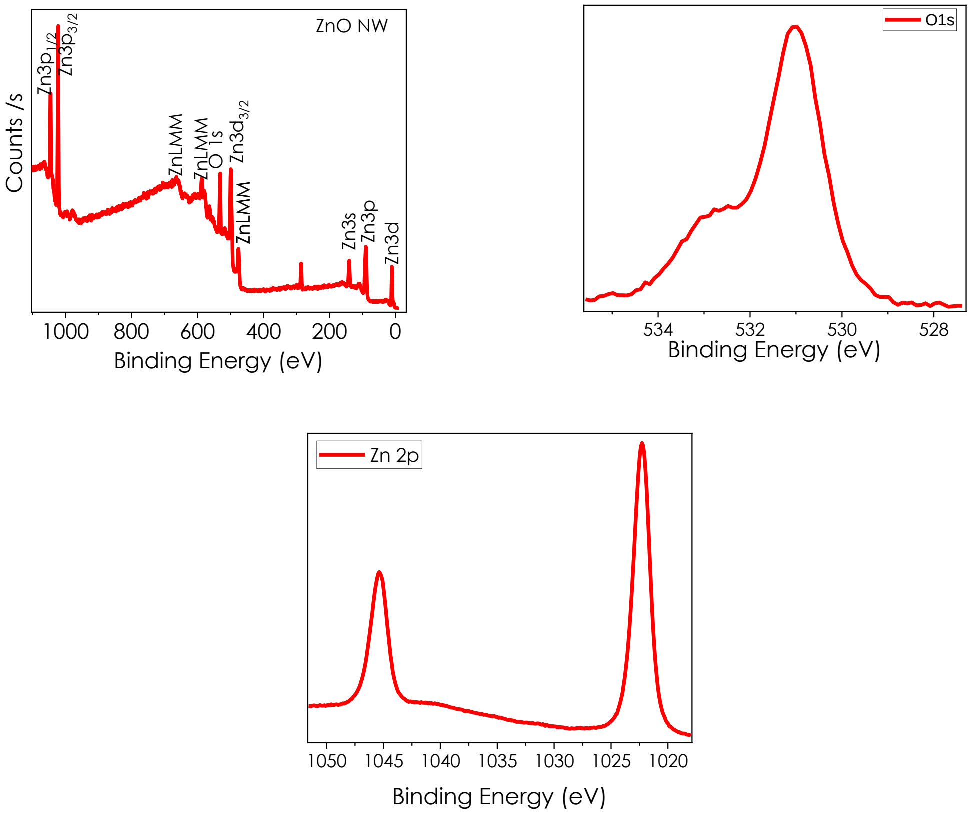

Figure S8. XPS spectra of ZnONW

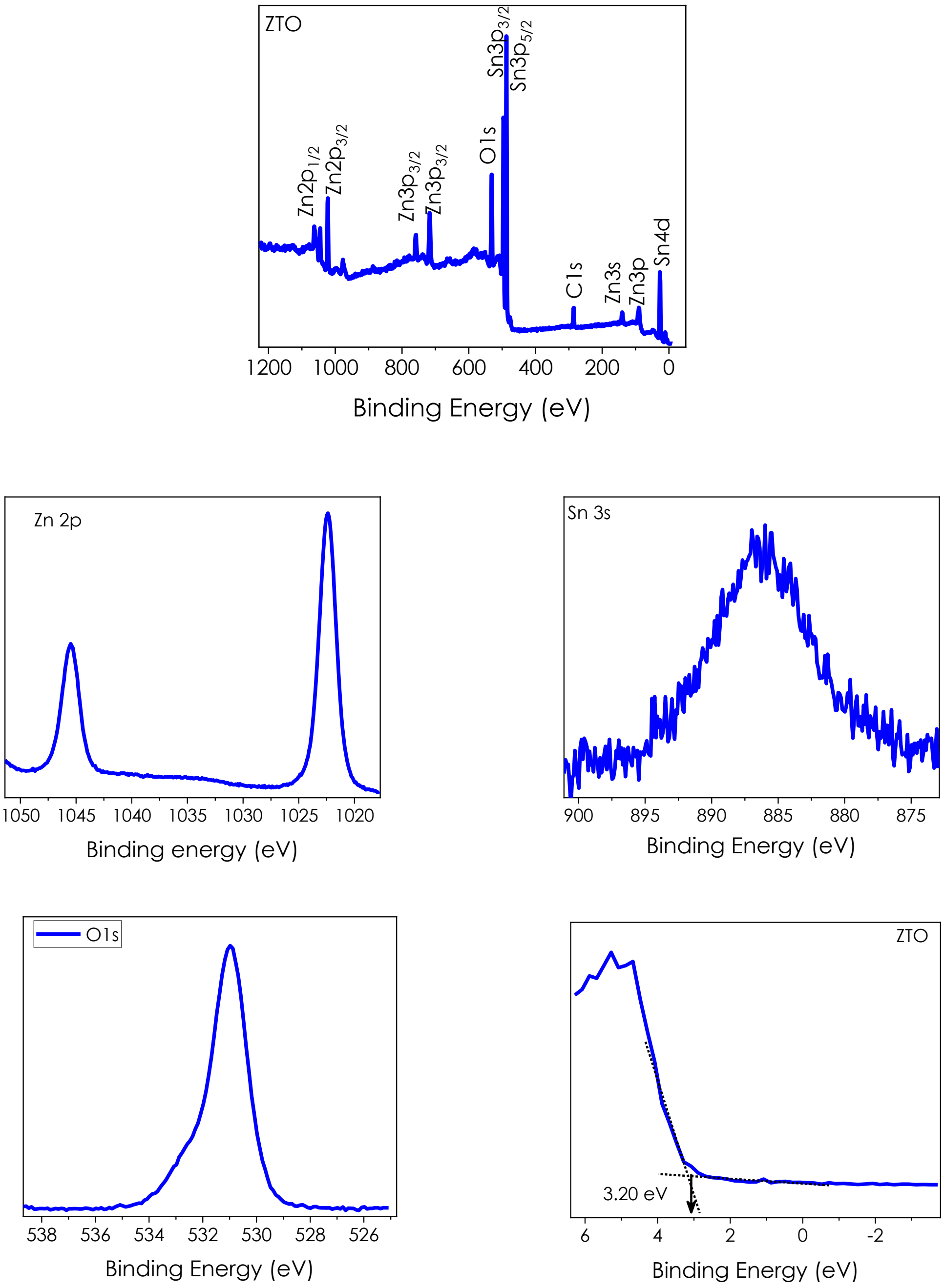

Figure S9. XPS spectra of ZTO

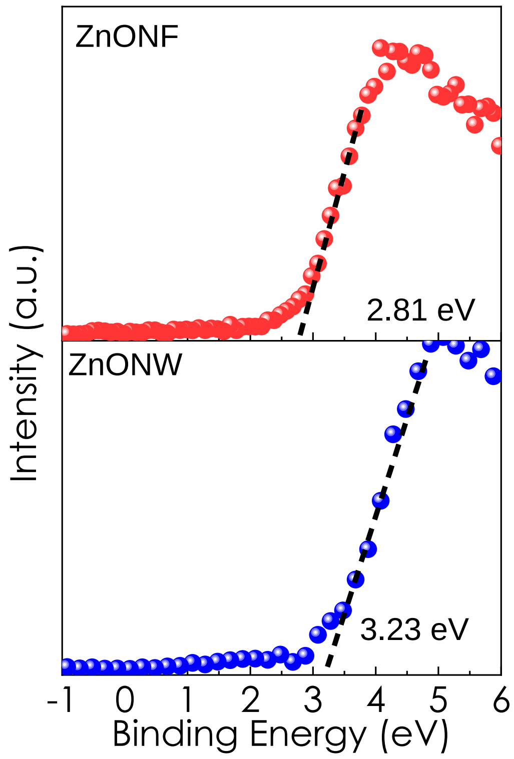

Figure S10. Valance band position of ZnO thin films.
